# Optimization of a Pain Model: Effects of Body Temperature and Anesthesia on Bladder Nociception in Mice

**DOI:** 10.1371/journal.pone.0079617

**Published:** 2013-11-05

**Authors:** Katelyn E. Sadler, Jarred M. Stratton, Jennifer J. DeBerry, Benedict J. Kolber

**Affiliations:** 1 Department of Biological Sciences and Chronic Pain Research Consortium, Duquesne University, Pittsburgh, Pennsylvania, United States of America; 2 Department of Neurobiology and Center for Pain Research, University of Pittsburgh, Pittsburgh, Pennsylvania, United States of America; University of Pecs Medical School, Hungary

## Abstract

Interstitial cystitis/bladder pain syndrome (IC/BPS) is a debilitating urological condition that is resistant to treatment and poorly understood. To determine novel molecular treatment targets and to elucidate the contribution of the nervous system to IC/BPS, many rodent bladder pain models have been developed. In this study we evaluated the effects of anesthesia induction and temperature variation in a mouse model of bladder pain known as urinary bladder distension (UBD). In this model compressed air is used to distend the bladder to distinct pressures while electrodes record the reflexive visceromotor response (VMR) from the overlying abdominal muscle. Two isoflurane induction models are commonly used before UBD: a short method lasting approximately 30 minutes and a long method lasting approximately 90 minutes. Animals were anesthetized with one of the methods then put through three sets of graded bladder distensions. Distensions performed following the short anesthesia protocol were significantly different from one another despite identical testing parameters; this same effect was not observed when the long anesthesia protocol was used. In order to determine the effect of temperature on VMRs, animals were put through three graded distension sets at 37.5 (normal mouse body temperature), 35.5, and 33.5°C. Distensions performed at 33.5 and 35.5°C were significantly lower than those performed at 37.5°C. Additionally, Western blot analysis revealed significantly smaller increases in spinal levels of phosphorylated extracellular-signal regulated kinase 2 (pERK2) following bladder distension in animals whose body temperature was maintained at 33.5°C as opposed to 37.5°C. These results highlight the significance of the dynamic effects of anesthesia on pain-like changes and the importance of close monitoring of temperature while performing UBD. For successful interpretation of VMRs and translation to human disease, body temperature should be maintained at 37.5°C and isoflurane induction should gradually decrease over the course of 90 minutes.

## Introduction

**Figure 1:**
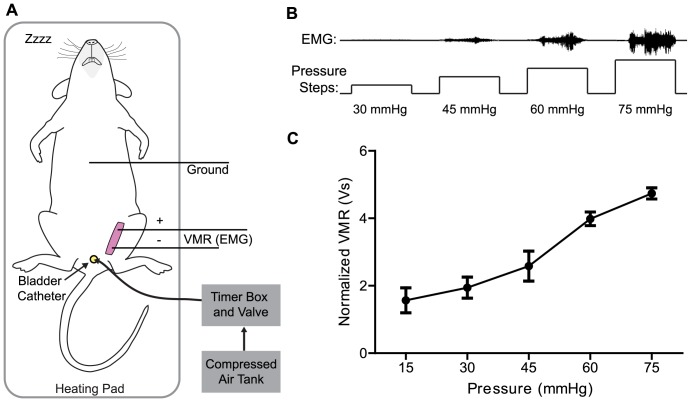
Visceromotor responses (VMR) from urinary bladder distension (UBD). (A) Schematic of UBD setup. Compressed air is delivered into bladder via urethral catheter. During distensions, electrodes in abdominal muscle record EMG. Temperature is maintained throughout the procedure using a battery operated heating pad and overhead lamp. (B) Example EMG traces during UBD. As pressure increases, electrical output from abdominal muscles increases congruently. (C) Example VMR from one complete set of distensions. During each set, the bladder is distended three times at each pressure. VMRs are normalized with 20 s pre-distension interval then averaged for each pressure.

Approximately 3–8 million people in the United States suffer from interstitial cystitis/bladder pain syndrome (IC/BPS), a debilitating condition characterized by increased urgency and frequency of urination as well as nocturia and general pelvic pain [1]. Despite years of research, the cause of IC/BPS remains elusive and treatment strategies are unable to provide complete relief to patients [2]. In order to study nervous system contributions to the condition, many animal models have been developed to mimic the pain and symptoms associated with IC/BPS. Traditionally, inflammatory agents such as cyclophosphamide [3], hydrochloric acid [4], acetone [5], mustard oil [6], lipopolysaccharide [7], [8], and infection with *E.coli* [8] have been used to induce sensitization of the bladder to model IC/BPS. However, since the exact inflammatory conditions accompanying IC/BPS are unknown, the non-specific bladder inflammation and contaminated urine in these models fails to mimic the real symptoms associated with the disease. An alternative or addition to these models is urinary bladder distension (UBD)[9]. In UBD, compressed air of a specific pressure is delivered to the bladder of a lightly anesthetized animal over a set period of time. Throughout the procedure wires in the superior oblique abdominal muscles record electrical activity known as the visceromotor response (VMR) (**Figure 1**). This activity is a reliable, reproducible measure of nociception [9]. To maintain VMR consistency between animals, confounding variables such as method of anesthesia induction and body temperature need to be taken into consideration. However, to date no study has systematically looked at the effects of these two factors on VMRs.

Isoflurane is an inhalable anesthetic that is commonly used in veterinary medicine due to its effortless induction, easy adjustability, and rapid recovery time [10]. Since metabolism of isoflurane is minimal in rodents, animals can be anesthetized by this compound upwards of 40 hours with the assistance of a ventilator [11]. Although the exact anesthetic mechanism of isoflurane remains unclear, it has been shown that the drug is able to bind to GABA receptors [12], glycine receptors [13], and glutamate receptors [14] among others. In UBD studies, isoflurane is used to sedate animals to a “partial” anesthetic level where a toe pinch induces a flexion response, but no vocalizations or ambulation; this typically occurs at 1% isoflurane [15]. However, the lowering of isoflurane to this suitable level can be accomplished by one of several induction methods. In these studies we examined the effects of isoflurane induction lengths on bladder distension-evoked VMRs. Due to the rapid expiration of isoflurane [16], we hypothesized that the method of isoflurane induction length would not induce significant differences in UBD-evoked VMRs.

In addition to the effects of anesthesia induction, body temperature is another variable that needs to be considered during UBD. Dramatic increases or decreases in temperature have long been known to alter respiratory rate, pulse, blood pressure, and cardiac output among other physiological parameters. To prevent these changes from occurring during UBD, animals' body temperatures must be constantly monitored and maintained at approximately 36.9°C, the average body temperature of *Mus musculus* [17]. While under general anesthesia, mice are not able to maintain their body temperatures due to quick metabolic rates and high body mass to surface area ratios [10]. Resulting hypothermia can greatly impact a number of physiological responses including pain sensation. Cold treatments are commonly used as a post-operative analgesic and have been found to significantly decrease pain following anterior cruciate ligament (ACL) replacement [18], total knee arthroplasty [19], and foot surgeries [20]. Similarly, cool skin temperatures significantly affect responses in the tail-flick [21] and formalin tests [22], two assays used to measure nociception in mice. In extreme cases whole-body cooling has been used to not only eliminate pain responses but also maintain animals in an anesthetized state after inhalable and injectable anesthetics have been metabolized. Dogs [23], cats [24], and monkeys [24], all enter a state of “cold narcosis” when their body temperatures are lowered to approximately 25°C. In the present study we hypothesized that decreased body temperature would result in a suppression of bladder-distension evoked VMRs. The overall goal of this study was to determine what factors, if any, affect the validity and reproducibility of VMRs. We found that decreased body temperature and the short isoflurane induction method significantly diminished UBD-evoked VMRs.

## Results

### Short isoflurane method affects VMR reproducibility

In order to achieve reliable, graded VMRs, animals are normally partially anesthetized for UBD; UBD can be performed in awake animals, however VMRs do not increase in a graded fashion when exceedingly noxious pressures are tested [9]. Due to its short induction time and the minimal levels at which it is metabolized, isoflurane is commonly used for this procedure [10]. Two common isoflurane induction protocols exist for use with UBD: a short method lasting approximately 30 min and a long method lasting approximately 90 min. Since both the long and short methods result in isoflurane levels at 1.0%, resulting VMRs are expected to be equivalent, however no study has yet to examine this prediction.

**Figure 2:**
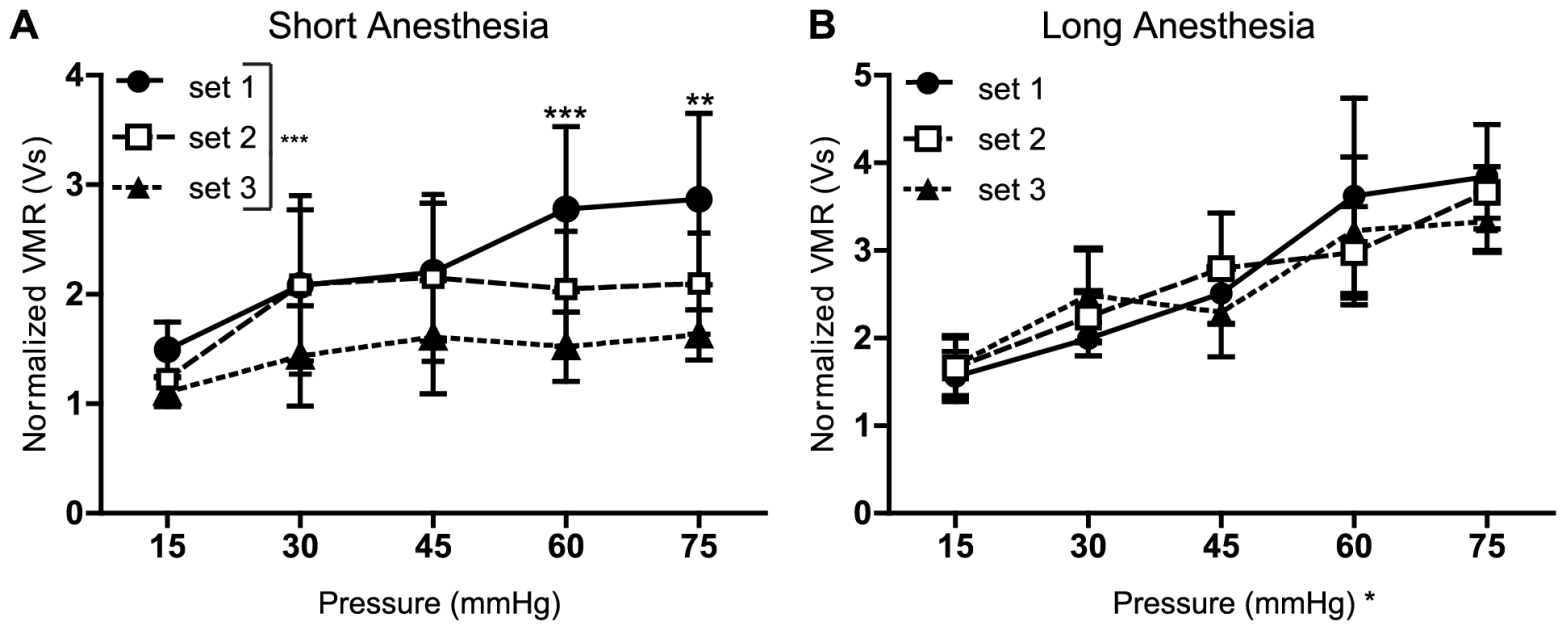
Isoflurane induction method affects VMRs. Short anesthesia method (A) was used to perform UBD (n = 6). As time progressed, VMRs steadily decreased. Two-way ANOVA revealed a significant difference between sets (***p<0.0001), but not between pressures (p = 0.7364). Bonferroni's posttest yielded differences between sets 1 and 3 at 60 mmHg (***p<0.001) and 75 mmHg (**p<0.01). When long anesthesia method (B) was used to perform UBD (n = 6), two-way ANOVA revealed no main effect of set number (p = 0.8751), but did yield a significant main effect of pressure (*p = 0.0436).

To test the effects of induction time, two cohorts of animals were sedated by either the long or short anesthesia method. Nociceptive responses were then assessed through three sets of distensions; each set lasted approximately 30 min and consisted of 15 distensions at graded pressures (15–75 mmHg). Surprisingly, in animals that underwent short anesthesia, there was a statistically significant main effect of distension set number (e.g. 1 vs. 2 vs. 3) on the UBD-evoked VMR; the graded quality of VMRs diminished in sets 2 and 3 (**Figure 2A**; two-way ANOVA, p<0.0001). Additionally, Bonferroni's posttest revealed statistically significant differences between set 1, which ended approximately 1 hr post-surgery, and set 3, which ended approximately 2 hr post-surgery, at 60 and 75 mmHg (60 mmHg, p<0.001; 75 mmHg, p<0.01). In animals that underwent long anesthesia set number did not have a significant main effect on VMR (**Figure 2B**; two-way ANOVA, p = 0.8751). However, as expected, pressure did have a statistically significant main effect on UDB-evoked VMRs; VMRs increased as bladder pressure increased (two-way ANOVA, p<0.05). Overall, these data suggest that a longer anesthesia induction produces a more stable VMR to bladder distension.

### Decreased body temperature dampens graded UBD responses

**Figure 3:**
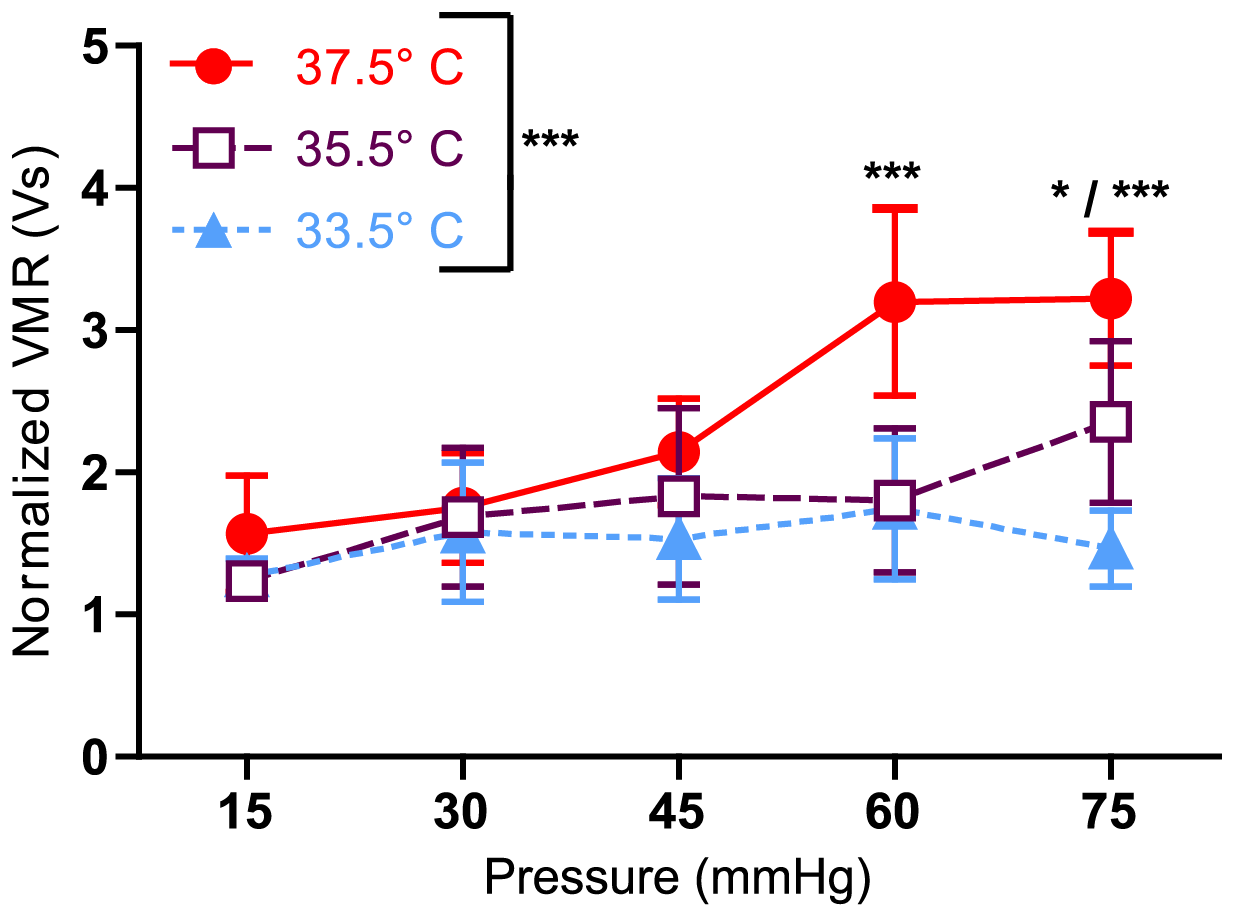
Animal's body temperature affects VMR. Following long anesthesia, animals (n = 6) were placed through three sets of graded distensions at 37.5, 33.5, and 35.5°C. Two-way ANOVA yielded a significant main effect of temperature (***p<0.0001), but not of pressure (p = 0.4536). Bonferroni's posttest revealed differences between 37.5 and 33.5°C at 60 mmHg and 75 mmHg (***p<0.001), as well as differences between 37.5 and 35.5°C at 60 mmHg (***p<0.001) and 75 mmHg (*p<0.05), and differences between 35.5 and 33.5°C at 75 mmHg (*p<0.05).

Decreasing an animal's body temperature can greatly affect many physiological characteristics, including pain sensation. In published accounts of UBD, body temperature is often reported as “monitored and maintained”, however no study has examined what effect lowering body temperatures might have on VMRs. We hypothesized that as an animal's body temperature decreases, VMRs will also decrease. To test this hypothesis, two cohorts of animals were sedated via the long anesthesia method. Nociceptive responses were then assessed with three distension sets, each lasting approximately 30 min and consisting of 15 distensions at graded pressures (15–75 mmHg). Each distension set was performed at a different temperature; set 1 was performed at 37.5°C, and sets 2 and 3 were performed at 33.5°C and 35.5°C, respectively, in order to avoid any confounding effects of anesthesia duration. Temperature had a main effect on VMRs (**Figure 3**) (two-way ANOVA long anesthesia, p<0.0001). More specifically, Bonferroni's post-test yielded significant differences between 37.5 and 33.5°C at 60 and 75 mmHg (p<0.001), between 37.5 and 35.5°C at 60 mmHg (p<0.001) and 75 mmHg (p<0.05), and between 35.5 and 33.5°C at 75 mmHg (p<0.05).

### Following decrease in body temperature, VMRs do not immediately return to original levels

**Figure 4:**
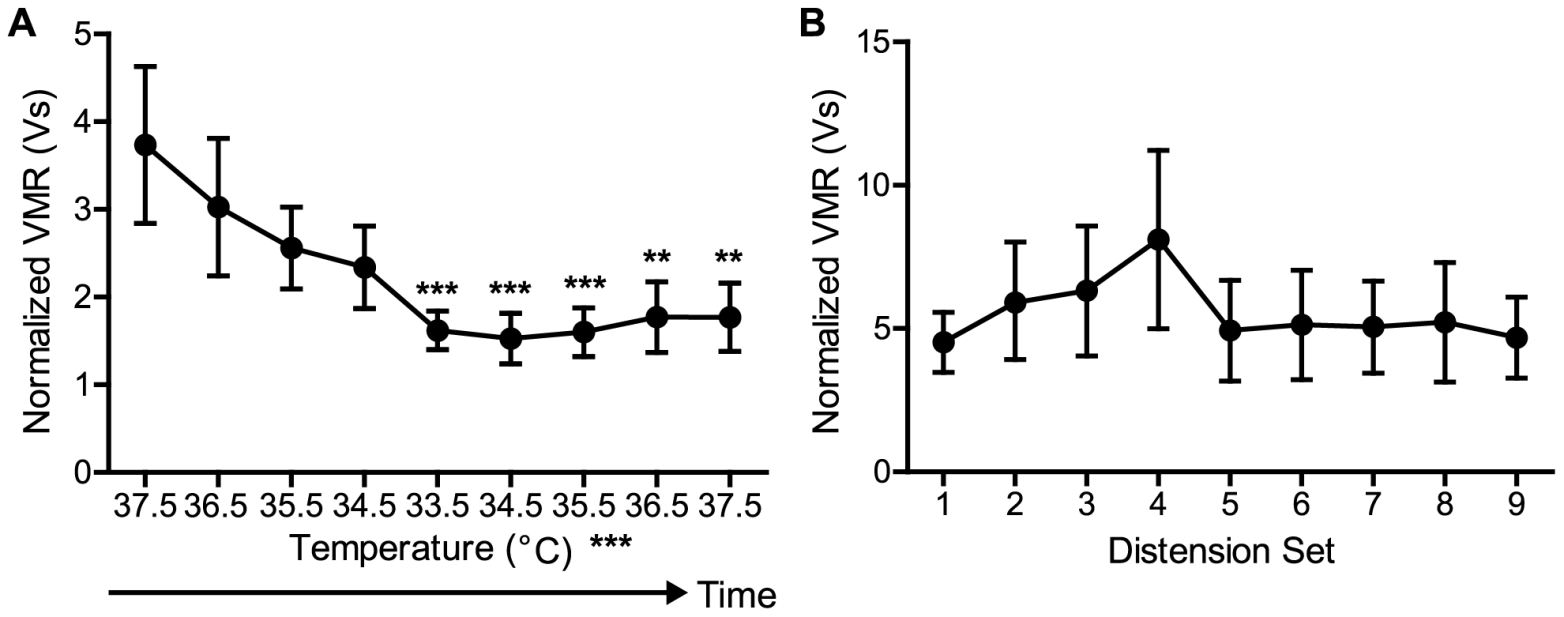
Following temperature drop, VMR does not return to baseline levels. (A) While performing UBD, animals' (n = 6) body temperature was decreased/increased by 1°C. One-way ANOVA revealed a significant main effect of temperature (***p = 0.0003). Additionally, the initial distension at 37.5°C was significantly different from the following distension sets: 33.5, second set at 34.5, second set at 35.5 (***p<0.0005), second set at 36.5, and second set at 37.5°C (**p<0.005). (B) When animals' (n = 6) body temperature was maintained at 37.5°C and the same UBD procedure was performed, one-way ANOVA yielded no difference between VMRs from any of the distension sets (p = 0.4081).

Following the experiments featured in **Figure 3**, it was apparent that a decrease in body temperature by even 2°C significantly impacted VMRs. However, it was unclear if this effect was reversible when body temperature was increased, or if it was a long-lasting phenomenon in partially anesthetized mice. To address this question, mice were put through nine distension sets at graded temperatures following long anesthesia. Each set consisted of three distensions at 60 mmHg (a noxious pressure); the first set was completed at 37.5°C, the second at 36.5°C and so on until the animals body temperature reached 33.5°C. Body temperature was then raised by 1°C and tested in a parallel fashion until the animal returned to 37.5°C. Overall, there was a significant main effect of body temperature on the UBD VMR (**Figure 4A**) (one-way ANOVA, p = 0.0003). The first distension set that significantly differed from the initial distension set at 37.5°C was that which occurred at 33.5°C (Bonferroni's posttest 33.5 vs. first distension at 37.5°C, p<0.0005). Furthermore, after body temperatures reached this lowest level, VMRs continued to be significantly reduced despite a return to the original body temperature of 37.5°C (**Figure 4A**) (second distensions at 34.5 or 35.5 vs. first distension at 37.5°C, p<0.0005; second distensions at 36.5 or 37.5 vs. first distension at 37.5°C, p<0.005). To ensure that this sustained decrease was not a confounding effect of anesthesia, this same experiment was repeated without any change in temperature. As seen in **Figure 4B**, there was no change in VMRs over time (one-way ANOVA, p = 0.4081) suggesting that the decreases in **Figure 4A** were strictly due to body temperature reduction.

### Decrease in body temperature reduces spinal pERK2 increase following UBD

Extracellular signal-regulated kinases 1/2 (ERK1/2) are activated via phosphorylation (pERK1/2) in the dorsal horn of the spinal cord following nociceptive stimulation [25]. Previous experiments from our lab and others have shown significant increases in pERK1/2 in the lumbosacral spinal cord following noxious bladder distension in mice [15], [26]. In order to gain a molecular understanding of the temperature-induced differences in VMRs, we investigated spinal pERK1/2 levels following UBD at normal and decreased temperatures.

**Figure 5:**
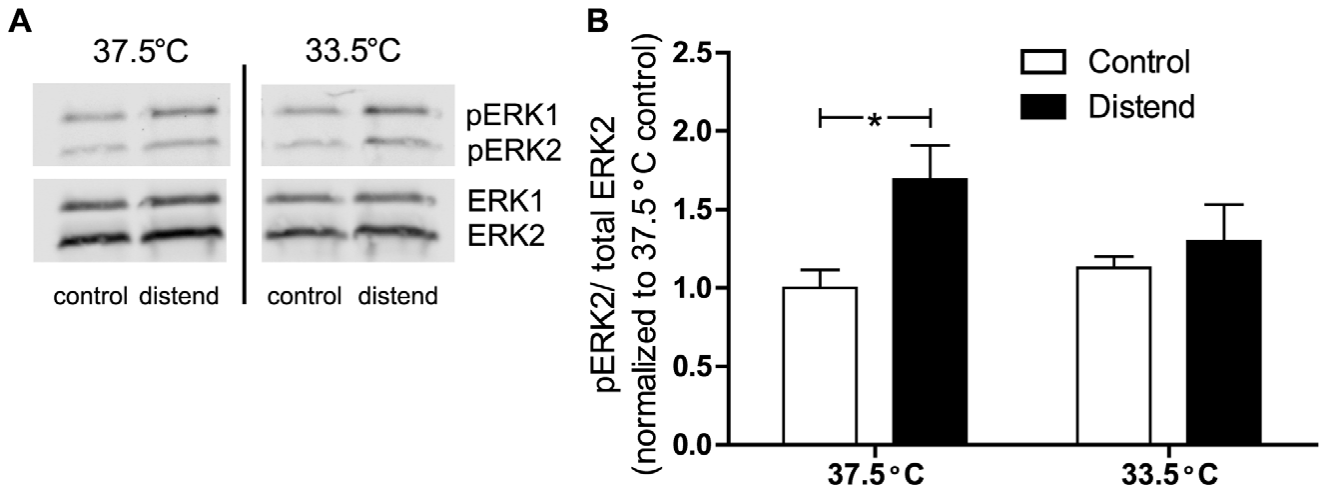
Decreased temperature dampens pERK2 difference in spinal cord. After long isoflurane induction, animals (n = 18) were distended 5 times at 75 mmHg or left undistended (control; n = 18). These manipulations occurred at 33.5°C for a subset of these animals (n = 9 distended and n = 9 undistended control). Animals were sacrificed 5 min post-distension and spinal cords were removed. Protein samples were run on a Western blot (A) then quantified. (B) pERK2 was divided by total ERK then normalized to the 37.5°C control group. Two-way ANOVA revealed a significant main effect of distension (p = 0.0120), however Bonferroni's posttest only reveals a significant difference between distend/control animals at 37.5 (*p<0.05).

Two different cohorts of animals were used for this experiment: one group that was maintained at 37.5°C and a second that was maintained at 33.5°C. After long anesthesia, one-half of each cohort was distended five times at 75 mmHg and one-half was left undistended (control). Following distension, spinal cords were removed and subsequently processed for pERK1/2 expression using Western blotting techniques (**Figure 5A**). In agreement with previous studies, there was a significant main effect of distention on pERK2 levels (**Figure 5B**; two-way ANOVA p = 0.0120). However, only 37.5°C distended mice showed a significant difference from 37.5°C control mice (**Figure 5B**) (Bonferroni post-test, p<0.05) [15]. Significant differences did not exist between baseline pERK2 levels in 33.5 and 37.5°C control animals. Additionally, there was no significant difference between 33.5°C distended and 33.5° control mice. No significant differences between groups were observed for pERK1 (data not shown).

## Discussion

Experimentally induced urinary bladder distension produces discomfort and prompts immediate pain-relieving action (i.e. urination) when performed in humans [27]. Since these symptoms are similar to those that naturally occur in patients suffering from IC/BPS, rodent models of UBD have been developed to more easily study the neurophysiological mechanisms underlying bladder pain [9], [28]. UBD can be achieved by inflating the bladder with fluid (saline, water, urine) or compressed air. When fluid is used, contraction of the distended organ can result in increased intraluminal pressure due to the low compressibility and high viscosity of fluids [29]. This increased distension pressure will result in further bladder contraction, thus initiating a positive feedback loop and unreliable VMRs. Conversely, air is highly compressible and has a low viscosity, allowing distension pressures to remain constant despite bladder contraction [29]. Versions of these models require that animals be lightly anesthetized throughout the procedure, however no study has yet to examine the effects of anesthesia induction or body temperature, both highly variable factors, on UBD results. A review of the literature in which UBD is employed reveals no specific comparison of isoflurane methods and either no mention of body temperature regulation or a generic phrase about, “thoroughly monitoring body temperature throughout the procedure” [8], [9], [15], [26], [30]–[32]. Our data, which shows that both anesthesia induction method and body temperature changes have an effect on VMRs, is vital to ensuring proper result interpretation and clinical translation.

Results from this study suggest that isoflurane methods can affect the reproducibility and validity of UBD-evoked VMRs. We found an effect of isoflurane time on VMRs when the short anesthesia induction method was used. **Figure 2A** demonstrates that distension sets ending 30 min post-surgery were significantly higher than those ending 1 hr and 1.5 hr post-surgery, despite vigilant monitoring of body temperature. These results were surprising; isoflurane is one of the most commonly used veterinary anesthetics since it is primarily removed through respiration and has low levels of metabolism. To our knowledge this is the first study in which a trend like this has been observed. Due to its prevalence in the clinical setting, previous studies have examined the effects of isoflurane duration on recovery parameters in humans. In children increased isoflurane duration resulted in longer times to extubation [33]. Similarly, the time it took rats to successfully pass the rotarod test following isoflurane exposure positively correlated with the length of time they had been anesthetized [34]. Although these tests were performed following isoflurane discontinuation, these results and our short isoflurane study (**Figure 2A**) results suggest that prolonged use of isoflurane may have sustained effects on the nervous system. However, this interpretation is in conflict with the long anesthesia method described here. If buildup of isoflurane was a uniform phenomenon, then we would have expected that animals given the long anesthesia method would exhibit lowered levels of UBD-evoked VMRs with increasing experimental time. In contrast to this prediction, UBD-evoked VMRs were stable for all three distension sets with the long induction anesthesia method. Furthermore, comparing the third distension following short anesthesia (**Figure 2A**) and the first distension set following long anesthesia (**Figure 2B**), one can appreciate a difference in the responses despite the fact that both of these sets were completed approximately 2 hr after isoflurane exposure began. One possible explanation for these data may be that the gradual step-down technique used in the long-anesthesia program allows for a modest amount of neural adaptation in response to isoflurane levels. Importantly, our data do show that following short anesthesia, only the first set of distensions produces an appropriate graded UBD VMR response. As a result, caution should be used when interpreting short anesthesia VMR data for experiments lasting longer than 1 hr.

In addition to anesthesia induction method, experimenters should also be acutely aware of body temperature during UBD testing. Results from this study suggest that body temperature affects the reproducibility and validity of VMRs. As seen in **Figure 3**, decreases in body temperature as small as 2°C result in significantly smaller VMRs. Furthermore, once an animal's body temperature falls to 33.5°C, VMR levels do not immediately recover even when body temperature is returned to 37.5°C (**Figure 4A**). The molecular explanation for these temperature differences may be partially attributed to pERK1/2 activation. Previous studies from our lab and others showed increased levels of pERK1/2 in the dorsal horn of the L6-S2 region of the spinal cord following noxious bladder distension [15], [26] and that this ERK1/2 activation was necessary for normal UBD-evoked VMR responses [26]. That same difference was confirmed in **Figure 5B** of the current study; at normal body temperature, significantly more pERK2 was found in animals that underwent bladder distension compared to their undistended control counterparts. However, when body temperature was lowered to 33.5°C, there was no difference between distended and undistended control animals (**Figure 5B**). Additionally, there is no difference in pERK2 levels between 33.5 and 37.5°C control animals. This suggests that decreased body temperature prevents the typical phosphorylation of ERK2 rather lowering the baseline amount of pERK2 present. Activation of ERK1/2 can lead to both central sensitization and increased excitability in the superficial dorsal horn through a variety of transcriptional and post-translational modifications including phosphorylation of NMDA receptor subunits [35], insertions of AMPA subunits into the plasma membrane [36], and involvement in long term potentiation [37]. However in the context of these studies, it is more likely that the rapid changes accompanying pERK1/2 inhibition of A-type potassium channels are responsible for the observable physiological changes [38]. In many studies, UBD is used in conjunction with pharmacological, electrical, or optogenetic methods of neural stimulation or inhibition. If these temperature effects are not taken into consideration, the smaller VMRs associated with decreased body temperature could be falsely attributed to the effects of one of the aforementioned manipulations.

Overall, these studies emphasize the importance of validating any anesthesia protocol when looking at nociceptive responses. Specifically, we suggest that when performing experiments in lightly anesthetized animals, the long isoflurane induction method should be used for studies lasting longer than 1 hr. In addition, these studies re-emphasize the importance of maintaining body temperature when performing experiments in anesthetized animals. In the case of UBD, multiple heat sources and vigilant monitoring techniques should be used to maintain body temperature at 37.5±0.5°C. Failure to do so will result in abnormally low VMRs that may be incorrectly interpreted. Optimizing isoflurane induction methods and temperature maintenance will ensure both the accuracy and reliability of VMRs as measures of bladder nociception and a more precise interpretation of experimental results.

## Materials and Methods

### Ethics Statement/Animals

All protocols were done in accordance with National Institutes of Health guidelines and were approved by the Institutional Animal Care and Use Committee at Duquesne University, Pittsburgh, PA (Protocol Number: 1110-14). Female C57Bl/6 mice aged 9–12 weeks were used for all experiments. Animals were housed on a 12 hr light/dark cycle with *ad libitum* access to rodent chow and water. All surgery was performed under isoflurane anesthesia (see below details) and efforts were made to minimize suffering throughout the experiment.

### Urinary Bladder Distension (UBD)

Urinary bladder distension (UBD) was developed as a tool for studying the mechanisms of IC/BPS and other urological conditions. In the laboratory setting, bladder distension produces discomfort and prompts patients to take immediate action, two feelings naturally experienced by those suffering from IC/BPS [27]. Since animals are not able to report these types of sensations to experimenters, the visceromotor response (VMR) has been validated as a measure of bladder nociception in rodents [9]. VMRs are a measure of electromyographic activity from the external oblique abdominal muscle that typically increase with increasing distension pressures (**Figure 1B**).

### Electromyogram recording electrode and bladder catheter implantation

Mice were anesthetized in an induction chamber and upon losing the righting reflex, were moved to a nose cone administering 2% isoflurane (vaporized with 95% O_2_/5% CO_2_). An incision was made in the skin of the lower abdomen, and two silver wires were implanted in the external oblique abdominal muscle. An additional grounding wire was laced through the skin overlying the chest cavity (**Figure 1A**). A lubricated 0.7 mm gauge, 14 mm long angiocatheter was placed into the bladder via the urethra. Animals were injected with 500 µL of 0.9% saline and artificial tears eye ointment was placed onto their eyes. Typical surgical time is 10 min.

### Testing parameters

After the short or long anesthesia method (see below for details), appropriate UBD VMR anesthesia levels were verified by the presence of the flexion reflex response in response to toe stimulation. If the reflex was absent, anesthesia was lowered another 0.125% for 10 additional min before testing resumed. Following a flexion response, compressed air was delivered to the bladder via a custom-made automated timer box (Washington University School of Medicine Electronic Shop, St. Louis, MO). Three initial distensions were done with 60 mmHg to verify standard VMRs and overcome the initial sensitization period [39]. Next, distension stimuli ranging from 15–75 mmHg were applied for 20 s, preceded by a 20 s pre-distension interval, and followed by a 1 min intertrial interval (ITI).

Throughout the entire experiment, electrical activity from the external oblique abdominal muscle was relayed in real time using a preamplifier (P5 Series, Grass Technologies) to the Spike2 data acquisition program (Version 7, Cambridge Electronic Design (CED)) via a CED module (1401 Plus, CED). These processed signals known as VMRs were exported to Igor Pro 6.22 software (Wavemetrics) where, using a custom script, they were subtracted from background, rectified and integrated over the 20 s stimulus. A similar script was used to analyze activity during the 20 s pre-distension interval. To normalize VMRs, all distention VMRs were divided by the smallest pre-distention interval value for an experiment. All VMRs performed at a single pressure were averaged for data analysis (**Figure 1C**). For all experimental testing, distensions were performed in triplicate for each pressure unless otherwise noted.

### Anesthesia induction methods

For all experiments in which the “short” anesthesia protocol was used, isoflurane levels were dropped to 1.0% immediately following surgery completion. After animals had been sustained at this level for 10 min, testing began. For all experiments in which the “long” anesthesia protocol was used, isoflurane levels were dropped to 1.5% immediately following surgery completion. Fifteen minutes later, isoflurane levels were decreased by 0.125% to 1.375%. This same reduction was repeated 3 more times over the next 45 minutes until isoflurane levels reached 1.0%. After animals had been sustained at 1.0% for 15 min, testing began.

For experiments examining the effects of anesthesia induction, animals underwent either the short or long induction method and were then put through a series of three distension sets. Each set lasted approximately 30 min and consisted of 15 distensions, three at each of the following pressures: 15, 30, 45, 60, and 75 mmHg. Distension triplicates were completed in a graded fashion from 15 to 75 mmHg. Immediately following the completion of one set, another was started. At the end of the third set, animals were sacrificed. Body temperature was maintained at 37.5 °C throughout all experiments.

### Body temperature methods

To test the effects of decreasing body temperature on VMRs, animals were anesthetized with the long anesthesia method. Throughout the experiment, animals' body temperatures were adjusted using a combination of overhead radiant lighting and a battery-operated heating pad (Kent Scientific) placed beneath the animal. A Right-Temp system (Kent Scientific) was used to monitor body temperature, blood oxygenation, and heart rate in real time. Body temperature was monitored using a subcutaneous probe. The subcutaneous probe was used to avoid possible visceral interference of a rectal temperature probe. In a control group of mice (no distention), no difference was seen between temperature at the subcutaneous site compared to a calibrated rectal probe (data not shown). These data are consistent with previous literature[40], [41] showing that subcutaneous temperature reading is a complementary approach to the more common use of rectal probes for temperature monitoring. Each animal was placed through a series of three distension sets. Each set lasted approximately 30 min and consisted of 15 distensions, three at each of the following pressures: 15, 30, 45, 60, and 75 mmHg. Distension triplicates were completed in a graded fashion from 15 to 75 mmHg. For the first set, the animal's body temperature was maintained at 37.5°C. At the completion of the first set, the animal's body temperature was lowered to 33.5°C, and after the temperature had stabilized for 5 min, the second set of distensions took place. The animal's body temperature was raised to 35.5°C for distension set 3.

In a second temperature-based experiment, animals were put through nine distension sets while their body temperature was being gradually decreased from 37.5°C to 33.5°C then returned to 37.5°C. Each set consisted of three distensions at 60 mmHg. The first set was performed after the animal's body temperature had been stabilized at 37.5°C for 5 min. Following this set, the animal's body temperature was lowered by 1° to 36.5°C and a second set was completed 5 min later. This same process was repeated for 35.5, 34.5 and 33.5°C. After the distension set at 33.5°C was complete, body temperature was raised by 1°C and distension sets were completed at each temperature until the animal returned to 37.5°C. As a control, this exact same experiment was repeated in a different cohort of animals, however their body temperature was maintained at 37.5°C for all nine distension sets.

### Western blotting

Levels of ERK1/2 and pERK1/2 in the spinal cord were analyzed by Western analysis. After catheterization, animals underwent long anesthesia induction (see above) until isoflurane levels reached 0.875%. Body temperature for half of the animals was maintained at 37.5°C. The remaining animals were maintained at 37.5°C until 15 min pre-distension when their temperature was lowered to 33.5°C. Experimental mice were distended 5 times at 75 mmHg (20 s distension, 1 min ITI, 20 s pre-distension interval). Control mice were catheterized but not distended. Spinal cords were removed by hydraulic extrusion 5 min after the last distension (or equivalent time for control mice), then the portions receiving afferent bladder innervation (L6-S1) were isolated and frozen on dry ice. This region began 2 mm caudal to the largest portion of the lumbar enlargement and contained the next 7 mm of tissue caudal to that point. All spinal cords were homogenized with ice-cold homogenization buffer (20 mM Tris, 1.5 mM EDTA, 1 mM Na_4_P_2_O_7_, 25 µg/mL aprotinin, 25 µg/mL leupeptin, 1X Sigma phosphatase inhibitors II and III, 100 µM PMSF), and then evaluated for total protein content using a BCA protein assay kit (Thermo Scientific, Rockford IL). 15 µg of protein from each spinal cord sample were separated on a 12% SDS polyacrylamide gel, then transferred to a nitrocellulose membrane. Blots were first blocked in Odyssey blocking buffer for 1 hr, then incubated with mouse anti-pERK1/2 (Cell Signaling, 1∶1,000) and rabbit anti-ERK1/2 (Cell Signaling, 1∶1,000) primary antibodies for 1 hr. Blots were washed and rinsed with TTBS, then incubated with goat anti-mouse AlexaFluor 680 (Invitrogen, 1∶20,000) and goat anti-rabbit IR 800 (Rockland, 1∶20,000) secondary antibodies for 1 hr. Blots were rinsed with TTBS then scanned on an Odyssey infrared imaging system. Using the Odyssey software (version 5.0), band densitometry was assessed and pERK1 or pERK2 amounts were normalized to respective total ERK1 or ERK2 amounts.

### Data Analysis

All statistical analyses were performed with GraphPad Prism 5 software. Data points are graphed as means ± SEM. For experiments with one independent variable and multiple groups, one-way ANOVA was to determine main effect. For experiments in which two independent variables were present, two-way ANOVA was used to determine main effects. Statistical significance was conferred to any test resulting in a p value less than 0.05. Bonferroni's *post hoc* tests were performed when a significant main effect was observed.
